# Demonstration and Methodology of Structural Monitoring of Stringer Runs out Composite Areas by Embedded Optical Fiber Sensors and Connectors Integrated during Production in a Composite Plant

**DOI:** 10.3390/s17071683

**Published:** 2017-07-21

**Authors:** Carlos Miguel Giraldo, Juan Zúñiga Sagredo, José Sánchez Gómez, Pedro Corredera

**Affiliations:** 1 Airbus Operations, S.L, Paseo John Lennon S/N, Getafe, 28906 Madrid, Spain; juan.z.sagredo@airbus.com (J.Z.S.); jose.g.sanchez@airbus.com (J.S.G.); 2 Materials Science, ETSII, Universidad Politécnica de Madrid, José Gutiérrez Abascal, 2, 28006 Madrid, Spain; 3 Instituto de Óptica (CSIC), 28006 Madrid, Spain; p.corredera@csic.es

**Keywords:** embedded optical fiber sensors, ingress-egress optical fiber, structural health monitoring in composite structures

## Abstract

Embedding optical fibers sensors into composite structures for Structural Health Monitoring purposes is not just one of the most attractive solutions contributing to smart structures, but also the optimum integration approach that insures maximum protection and integrity of the fibers. Nevertheless this intended integration level still remains an industrial challenge since today there is no mature integration process in composite plants matching all necessary requirements. This article describes the process developed to integrate optical fiber sensors in the Production cycle of a test specimen. The sensors, Bragg gratings, were integrated into the laminate during automatic tape lay-up and also by a secondary bonding process, both in the Airbus Composite Plant. The test specimen, completely representative of the root joint of the lower wing cover of a real aircraft, is comprised of a structural skin panel with the associated stringer run out. The ingress-egress was achieved through the precise design and integration of miniaturized optical connectors compatible with the manufacturing conditions and operational test requirements. After production, the specimen was trimmed, assembled and bolted to metallic plates to represent the real triform and buttstrap, and eventually installed into the structural test rig. The interrogation of the sensors proves the effectiveness of the integration process; the analysis of the strain results demonstrate the good correlation between fiber sensors and electrical gauges in those locations where they are installed nearby, and the curvature and load transfer analysis in the bolted stringer run out area enable demonstration of the consistency of the fiber sensors measurements. In conclusion, this work presents strong evidence of the performance of embedded optical sensors for structural health monitoring purposes, where in addition and most importantly, the fibers were integrated in a real production environment and the ingress-egress issue was solved by the design and integration of miniaturized connectors compatible with the manufacturing and structural test phases.

## 1. Introduction

The integration of optical fibers sensors into composite structures for Structural Health Monitoring (SHM) purposes by embedding them inside the lay-up is not only actually one of the most attractive solutions contributing to the realization of smart composite structures [1,2,3,4], but also the optimum integration approach that insures the maximum protection and integrity of the fibers under the operating conditions. Nevertheless this intended integration level still remains an industrial challenge since there is today no mature optical fiber integration process in composite plants matching all the necessary requirements such as a robust ingress-egress optical fiber method, a simple but reliable optical fiber placement and connection, compatibility with the composite production process or the complete assurance of the fibers’ and their connectors’ resistance to the environmental and the operational conditions [5,6].

The two main objectives of this article are to present firstly the methodology followed for the integration of optical fiber sensors in the automatic tape layout (ATL) of the Airbus Illescas Production Plant, including how the critical point of the ingress-egress of the optical fibers is solved by the design and integration of miniaturized optical connectors compatible with the manufacturing process and also the structural tests phases, static and fatigue cases, that the specimen was subjected to. The second objective is to address the analysis of the optical fiber results in order to demonstrate the performance and the consistency of the experimental measurements of the embedded sensors by the correlation with conventional strain gauges in those cases make sense, or by the curvature and the load transfer analysis in the bolted area. 

The development started with the interest of the Stress Staff in monitoring the mechanical strain in the bolted and hidden composite area of a stringer run out test specimen completely representative of the root joint area of the wing lower cover of a real aircraft. The specimen comprises a flat skin panel strengthen by a co-bonded T-shape stringer and covered and clamped to a metallic triform and buttstrap. The metallic plates on both sides of the specimen prevent the installation of any strain gauges on the composite area and led initially to the use of conventional strain gauges on the metallic surfaces as the only way to monitor this area. Thereafter, a capture requirement process was initiated to identify every task in the serial manufacturing process that could be impacted by the integration of the fibers. The main requirements are synthetized in the Section 3 of this document. Once the capture was finished, the work consisted of the preparation and development of the methodology and tools to integrate the fibers and connection devices inside the normal Production cycle. The fiber sensors were integrated into the composite skin during ATL, and between the stringer foot and control thickness material by a secondary bonded process. The integration of homemade miniaturized optical connectors has ensured a robust ingress-egress with no risk of fiber breakage from the trimming process of the specimen up to the working tasks related to the installation of the specimen in the test rig. The interrogation of the fibers during the two static tests, before and after the fatigue campaign, has also enabled demonstration of the resistance of the designed connector against fatigue conditions. Finally, the analysis of the strain results demonstrate the good correlation between fiber sensors and electrical gauges in those locations where they are installed nearby, and the curvature and load transfer analysis in the bolted stringer run out area enables demonstration of the consistency of the fiber sensors measurements.

## 2. Fiber Bragg Grating Sensors and Integration Methods in Composite Parts

**Figure 1:**
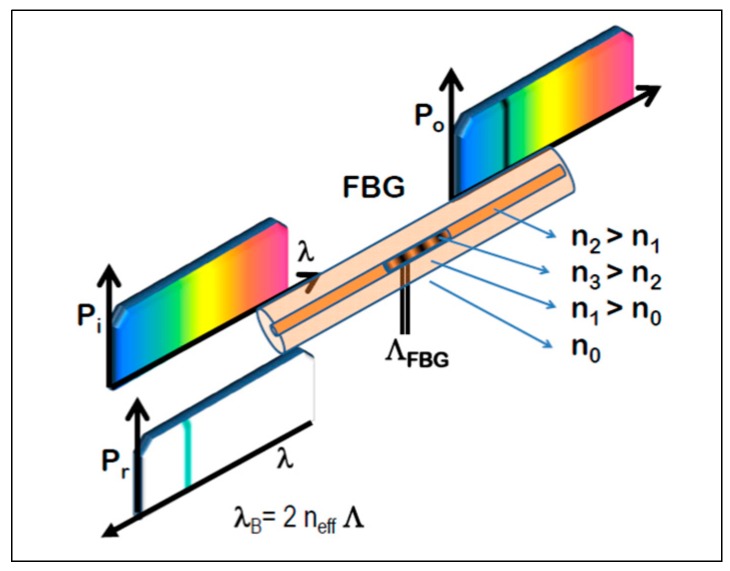
Physical principal of Fiber Bragg Grating sensor.

Fiber Bragg Grating (FBG) sensors were the optical fiber sensors used in the described application. A FBG is a local sensor typically used for the measurement of strain and/or temperature sensing written in the core of a single mode optical fiber. Basically it consists of a permanent periodic perturbation of the refractive index along a short length [7]. When the light from a broadband source interacts with the grating, a single wavelength is reflected back, whereas the rest of the signal is transmitted (Figure 1).

The central wavelength of the reflected component (λ_R_) satisfies the Bragg condition:

λ_R_ = 2nΛ (1)

where n is the index of refraction and Λ the period of the index of refraction variation. Both parameters are intrinsically sensitive to temperature and strain (to a lesser extent the parameters are also sensitive to pressure, and even humidity if the fiber coating is hydroscopic [8,9]) and produce a wavelength shift when strain and/or temperature are applied.

The change of wavelength of an FBG due to strain and temperature can be approximately described by the following equation [10,11]:

Δλ(ε,T)/λ_o_ = (1 − p_ε_)ε + (α + ξ)ΔT (2)

where Δλ is the wavelength shift, λ_o_ is the initial wavelength, p_ε_ is the strain-optic coefficient, ε is the strain experienced by the grating, α is the thermal expansion coefficient of the grating, and ξ is the thermo-optic coefficient describing the change in refractive index with temperature.

In the main telecommunications transmission frequency, the equation can be simplified to:

∆λ(ε,T) = S_ε_ × ε + S_T_ × ΔT (3)

where S_ε_ is the wavelength/strain sensitivity, approximate value of 1.2 pm/μstrain, S_T_ is the wavelength/temperature sensitivity, roughly 10 pm/°C. 

Since FBGs respond to strain and temperature, when both parameters are acting simultaneously, the output of the sensor is the combination of the two factors and a temperature compensation method needs to be applied for the proper strain or temperature measurement to decouple the different effects [12,13]. 

FBG technology enables several gratings to be written along a single optical fiber in the locations to be monitored and therefore producing a quasi-distributed sensing system. Optical fiber sensors can be applied in two ways in composite structures: surface bonded or embedded inside the composite layup during production phase [14,15]. Surface bonded methodology, although requiring special training and skills, is very similar to the surface preparation and adhesives used for the bonding process of classical electrical strain foils. Embedded sensors have to be considered a more complex process requiring the implementation of the sensors during production time in the stacking clean area. This process requires a detailed analysis of the production tasks in order to develop an integration process compatible and effective inside the Production cycle.

The next section addresses to the two key elements for the successful integration of the fiber in the composite parts, the fiber itself and the ingress-egress, and how these elements have to be selected and designed to be consistent with manufacturing and the operational phase of composite parts.

## 3. Embedding FBG Sensor in Composite Parts

Two group of tasks have to be distinguished in the design process of embedding optical fiber in the structural composite parts. Firstly the group of tasks addressed to prepare and integrate the fiber inside the layers of the host composite material, and secondly the development of a solution for the fiber ingress-egress to ensure proper protection and connection.

### 3.1. Embedding the Fiber

The main aspects that were considered when embedding optical fibers in our composites structure application are:

(a) The selection of the fiber and specially the coating of the fiber which determines the resistance to temperature, handling or repair methods. In our application, single mode fiber coated by ORMOCER (an inorganic-organic hybrid polymer developed at the Fraunhofer ISC) [16] was selected.
(b) Intrusiveness of the fiber inside the material. This is described by the lack of homogeneity inside the composite material in the vicinity of the embedded optical fiber and can cause the local bending on the reinforcement carbon fiber or the accumulation of resin close to the fiber. If no special care is taken, the intrusiveness effect can degrade some structural properties of the composite material. The proper selection of the fiber diameter, the relative direction between the reinforcement and optical fiber, the depth and location of the fiber in the part thickness are some of the most important issues to consider in order to make negligible any structural effect due to the embedded fiber. In our case, the fiber was positioned parallel to the reinforced material and two layers above the bottom surface.
(c) Fiber routing. The fiber routing was done in accordance with the required monitoring locations, measurement directions, the location of the drillholes, and connections.

### 3.2. Ingress-Egress Solution

The ingress-egress of the optical fiber inside composite material parts is the transition area between the embedded fiber in the composite and the external fiber optic to be connected to the interrogator unit. This transition area can be in principle located in the edge of the structure or through the surface, depending essentially on the boundary conditions of the part and the manufacturing constraints. The main requirements applying to the ingress-egress solution adopted in our application scenario are summarized below:

(a) Material resistance of the connection device to fulfill with criteria such as:
  - Resistance to temperatures ranging from 180 to 220 °C during composite curing conditions to −45 °C during in-service operations.
  - Compatible thermal expansion with the composite part during and after the process of curing the composite structure.
  - Compatibility with aeronautical environment conditions such as mechanical loads, changes of humidity, pressure, corrosive environments, crash or vibrations.
(b) Streamlined outer shape to avoid the formation of porosities and holes around the embedded connection device.
(c) Tightness is extremely important, not only for the in-service life of the connection device, but especially during the process of curing the composite structure itself.
(d) Minimum size device. This is actually one of the main shortcomings of the solutions available today; connectors are too large to be embedded inside the material compromising the structural integrity of the part in this area.
(e) Compatibility with the typical trimming process of composite parts. The adapted solution enabled the edging operation without damaging the integrity of the connecting device.
(f) Robust methodology providing the maximum fiber survivability, repeatability in optical performance features such as insertion loss or return loss, and locking mechanics ensuring a minimum number of plugs. The integrated connector in the presented application was robust enough to perform stable measurements in the embedded fiber during all structural test cases, before and after the fatigue campaign. The average insertion loss was 1 dB and stable over the complete measurement process.

## 4. Experimental Set-Up 

### 4.1. Description of the Test Specimen

**Figure 2:**
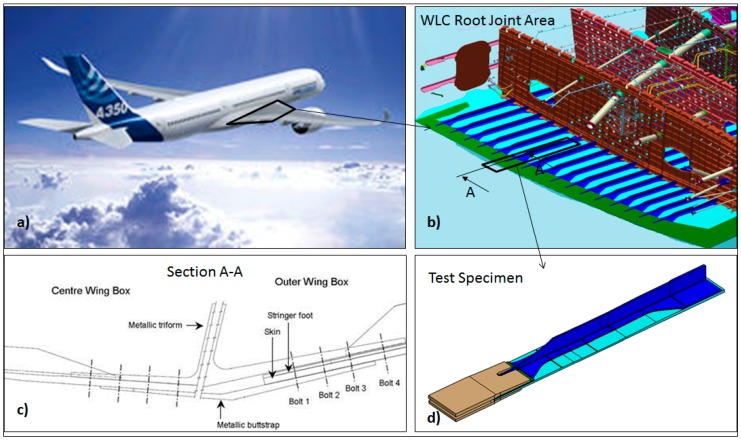
(**a**) Full aircraft, (**b**) Wing Lower Cover Root Joint Area, (**c**) Section A-A of Root Joint Area and (**d**) Test specimen

The test specimen consisted of a run out skin-stringer configuration representative of the wing lower cover in the area where it is jointed to center wing box (Figure 2). The specimens were manufactured at the Airbus Production Plant according to the following sequence: firstly the automatic tape layup & curing of the skin, secondly the co-bonding of the stringer to the skin, thirdly the secondary bonding between the thickness control material (TCM) and the stringer foot, and finally the clamping of the triform and buttstrap at the run-out end. 

A total of three specimens were instrumented with embedded optical fibers. The first one was the calibration specimen, normally used only to determine the level of impact energies that are necessary to produce barely visible impact damage (BVID) in composite structures. The manufacturing process of this first specimen served to determine the methodology for embedding the fibers and connectors inside the material. The other two specimens, referred hereinafter as the test specimens 1 and 2, were subjected to mechanical tests in the test facilities. Each specimen was introduced in a test rig and anti- buckling system to ensure the proper loads and stress distribution. The structural tests were performed at room temperature.

### 4.2. Description of the Integration of the Optical Fibers

FBG sensors were installed in the test specimen under two scenarios: embedded between skin layers during production time, and surface bonded on the stringer foot and then hidden by the secondary bonded thickness control material.

#### 4.2.1. Embedded FBG Sensors between Skin Layers during Production Time

**Figure 3:**
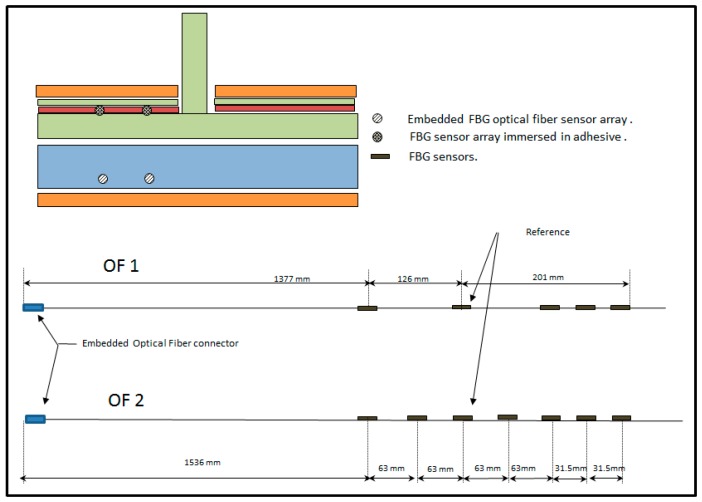
FBG arrays configurations and locations.

The first step consisted of the preparation of the FBG sensor arrays according to the dimensions of the part, the specification of the positions of the sensors in the structure and the requirements defined in Section 3.1. Two configuration arrays were initially prepared, named as 1 and 2 and depicted in the Figure 3.

The FBG sensors arrays were written during the drawing process of the fiber (DTG) [17] by FBGS company. The main features of the arrays are compiled in Table 1.

**Figure 4:**
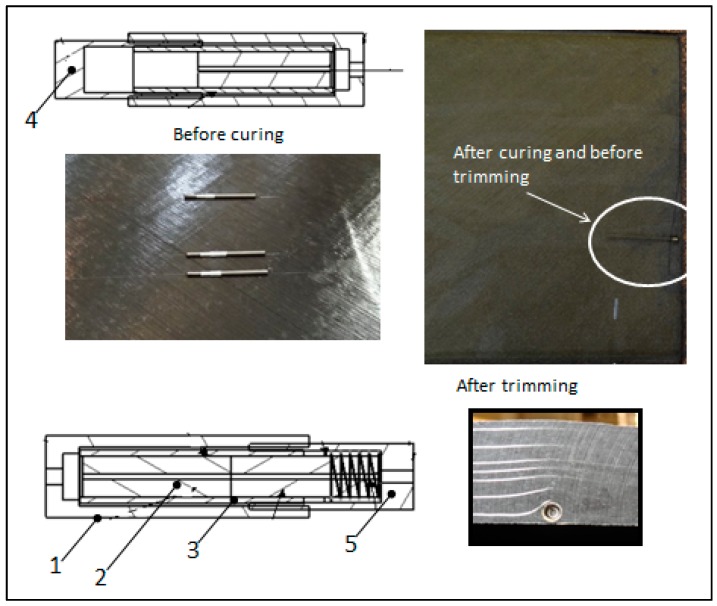
Optical fiber connector solution.

The second step was the assembly of the fibers to a special trimmable connector device. This trimmable connector was specially designed and manufactured in order to be compatible with all the steps in production and comprised the following elements (Figure 4):

(a) A first connecting element (1) embedded in the composite material and located inside a resistant compartment. This first element comprises a precisely positioned optical ferrule (2) connected to a sleeve guide (3) for directing and centering with the optical fiber core coming from external interrogator unit.
(b) A protective element (4) that makes the first connecting element perfectly sealed during the manufacture process, and thus preventing the intrusion of resin into the first connection element. This protection is withdrawn once the structure is cured and before the trimming operation.
(c) A second connection element (5) that is fit to the first connection element after removing the previous protective element when the sensors need to be interrogated.
(d) Finally a mechanical protection joined to the second connecting element, and whose purpose is to protect mechanically the first and second connection elements in a way that any mechanical effort (vibration, shock, etc.) is absorbed by this protective element.

The fibers with the connectors were installed in production time during the automatic tape lay-up process (ATL). The precise location of the fibers and sensors inside the laminate were done taking as reference two special marks purposefully made on manufacturing tools, one in the axial direction and the other one transversally. These marks were in turn referenced to an additional permanent mark done on all the FBG arrays. After the location of the fibers on the pre-preg, the rest of the layers were laid up to complete the skin stacking. Finally the fresh skin was placed in the autoclave to complete the cure process according to the corresponding cycle conditions.

**Figure 5:**
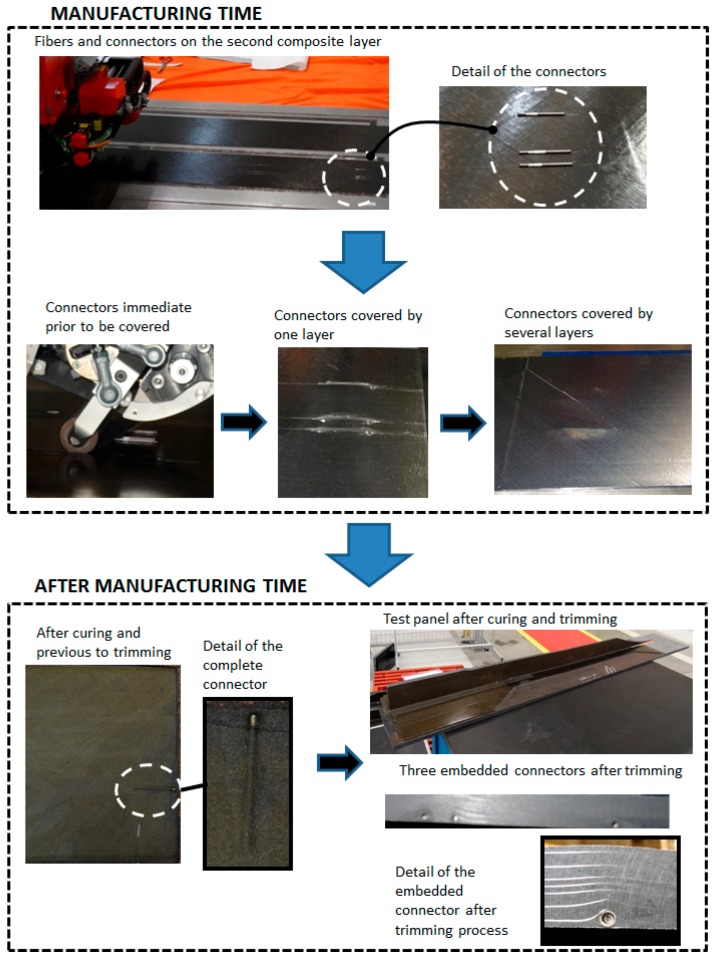
Optical fiber connector at different times during the manufacturing process.

Figure 5 shows the location of the optical fiber connectors during different times of the process sequence; firstly on the lay-up, secondly before being covered by composite layers, thirdly covered by layers, fourthly after curing and finally after trimming process.

#### 4.2.2. Surface Bonded Fiber Sensors on the Stringer Foot

Two FBG fibers were surface bonded on the stringer foot. The configuration of the FBG sensors coincides with the configuration 1 defined previously for the embedded fibers. After the bonding process they were covered by a TCM and finally sandwiched between metallic butt straps.

Initially this configuration of the fibers -sandwiched between stringer foot and buttstraps- presented two important issues to solve, on the one hand the possible breakage of the fiber during manufacturing tasks when installing the TCM on the transition points of the fiber, and on the other hand there was also a risk of distortion of the FBG peaks by the transversal stress once the set is complete assembled. In order to deal with these two issues and to ensure the effectivity of the fiber sensors in the test, a special solution was prepared and accepted by Stress and Manufacturing.

It consisted of the preparation of two precise grooves on the stringer foot during the curing time of the stringer to locate the fibers. These grooves were made by means of two calibrated metallic wires that were selected by the results in previous manufacturing trials done in the Composite Lab taking into account the diameter, material and facility to remove them after the process. Once the grooves were formed, the FBG arrays were installed and bonded inside, then the TCM were bonded to the stringer foot and finally the metallic buttstrap and triform were adjusted, drilled and fitted by the fasteners. External fibers were protected by optical fiber cable.

**Figure 6:**
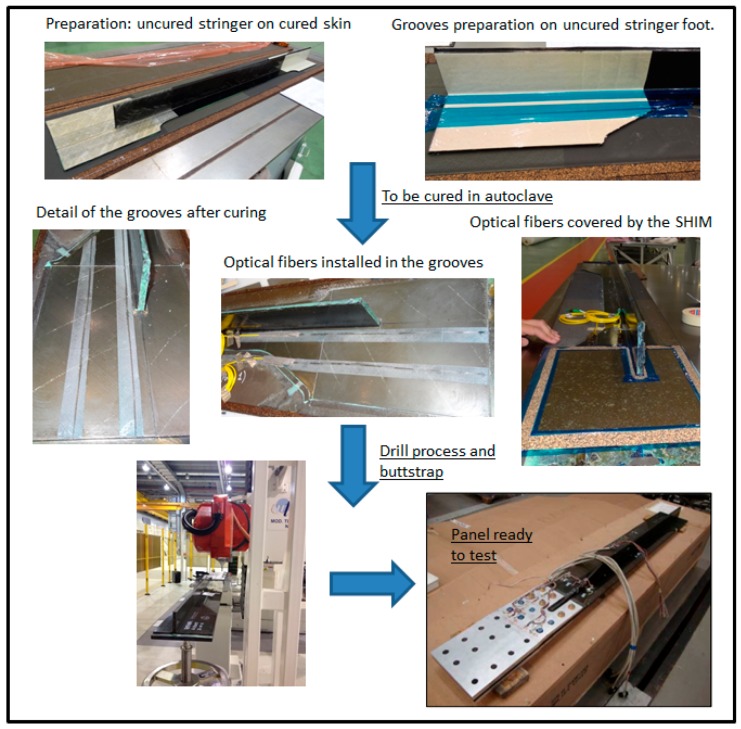
Installation of FBG arrays on the stringer foot.

Figure 6 summaries the complete process followed for the installation of these two fibers on the stringer foot.

### 4.3. Classical Strain Gauges on the Metallic Plates and Stringer Foot Surface

**Figure 7:**
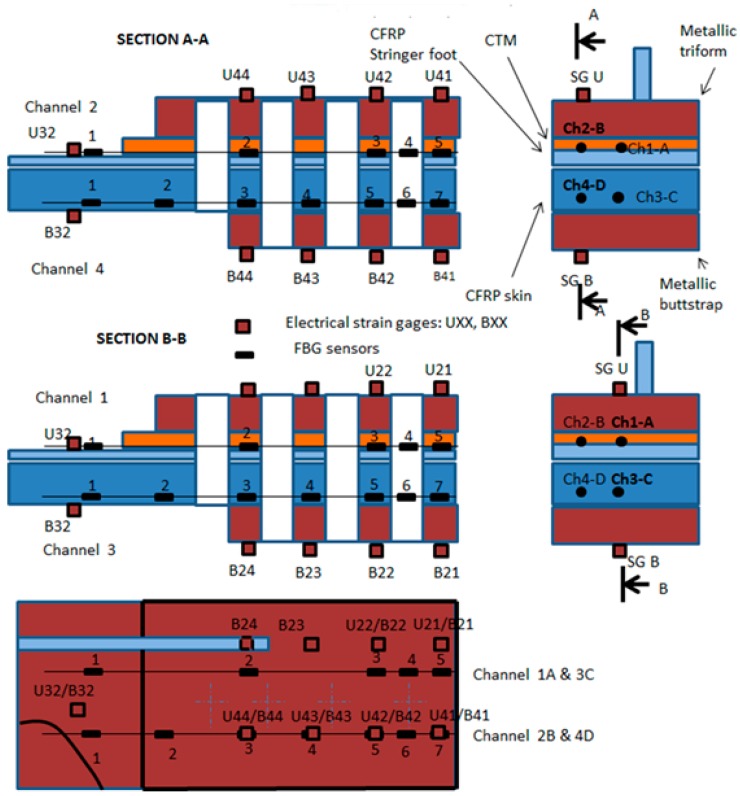
Locations of the strain gauges and FBG sensors.

In addition to the described optical fiber sensors, strain gauges were also surface bonded on the metallic triform, buttstrap and on the composite skin. These sensors were the instrumentation used by Stress to ensure and control the proper introduction of the load in the specimen during the structural test. These sensors will be also referred to in the results and discussion as the comparative technology used to verify and validate the optical fiber measurements. Figure 7 displays the sections and the plan view with the locations of the strain gauges and FBG sensors in the stringer run out area.

## 5. Results

During the structural test, the test specimens 1 and 2 were subjected to the following load sequence:

(a) Static cases of tension and compression up to Limit Load (LL) at 650 kN and −300 kN respectively.
(b) Fatigue test campaign. This campaign was composed of 10 fatigue blocks reaching a maximum load of 650 kN and minimum load of −140 kN.
(c) Final tension up to Limit Load (LL) and compression tests beyond Ultimate Load (UL) up to −610 kN.

For the specimen 1, the test cases acquired with optical fibers were, before fatigue, tension and compression up to Limit Load (LL), and after fatigue, compression up to Ultimate Load (UL). For the specimen 2, the acquisitions were done after the fatigue phase and corresponded to tensile static test up to UL and compression static test up to load higher than UL.

The sampling rate for the FBG interrogation unit was 20 Hz and was selected to be compatible with the strain gauge acquisition rate.

The next plots from Figure 8, Figure 9, Figure 10, Figure 11, Figure 12, Figure 13, Figure 14, Figure 15 and Figure 16 correspond to some of the different tests acquired by the optical fiber sensors and are referred in the discussion of the results. These graphs correspond to specimen 1 as it is the data selected for the analysis.

## 6. Discussion

The analysis of the test data described in this article is based on the strain gauge and optical fiber sensors that can be compared considering their location in the specimen. The specimen that has been used for the correlation and analysis is Specimen 1 which has been loaded up to Limit Load in Tension and Compression and finally up to a load level close to Failure Load in Compression. The following sensors have been identified for quantitative comparison:

- Strain gauge U32 as the average between A1 and B1 FO sensors
- Strain gauge B32 as the average between B1 and D1 FO sensors

### 6.1. U32 and B32 Correlation

The strain gauges U32 and B32 are not vertically located above any OF sensor, but they are located between the two OF lines as shown in Figure 7. Therefore, in order to analyze their correlation, the average between the corresponding OF sensors has been calculated. The fact that OF lines C and D are not placed exactly on the specimen lower surface, as they are covered by two carbon fiber layers, has been disregarded for this analysis. The correlation that has been checked is:

- U32 vs. Average (A1,B1)
- B32 vs. Average (C1,D1)

The correlation has been performed at several load steps of the following test sequences:

- Tension LL: 650 kN
- Compression LL: −300 kN

Additionally, a third test sequence corresponding close to the failure of the specimen in compression has been checked:

- Compression Failure: −610 kN

The data used in the correlation corresponding to the Limit Load in Compression and Tension is presented in Table 2.

The average between the OF sensors and the correlation with the strain gauge data is provided in Table 3 for the Limit Load test sequences.

**Figure 17:**
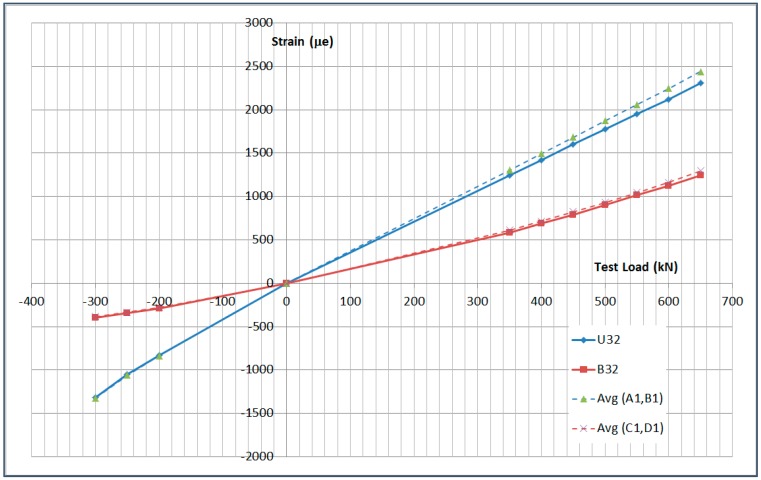
Limit Load Test Data Comparison.

**Figure 18:**
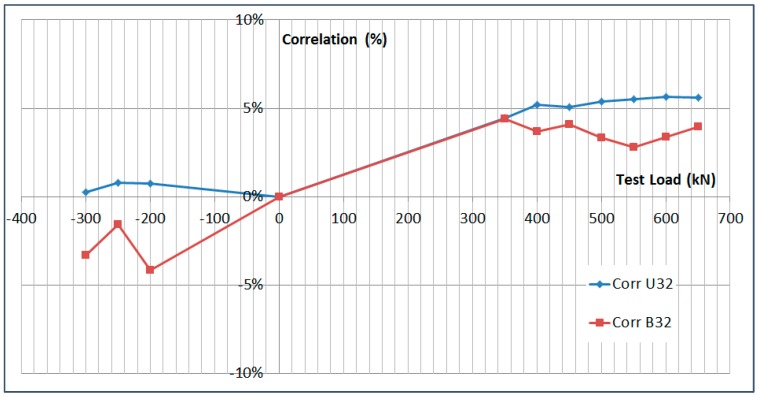
Limit Load Test Data Correlation.

The previous data is shown graphically in Figure 17 and Figure 18.

The data used in the correlation corresponding to the Failure Load in Compression is presented in Table 4 and Table 5.

**Figure 19:**
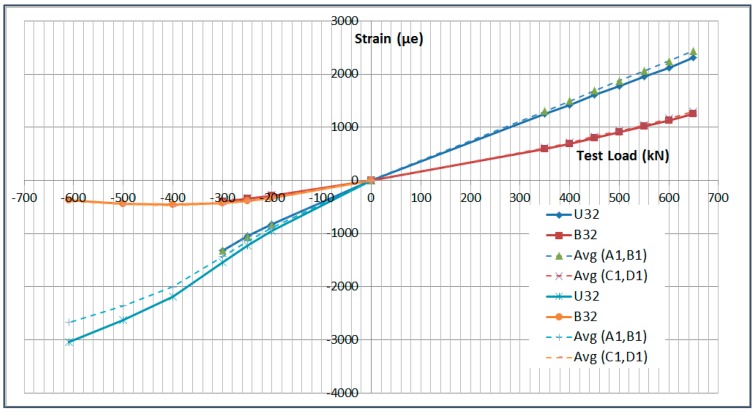
Failure Load Test Data Comparison.

**Figure 20:**
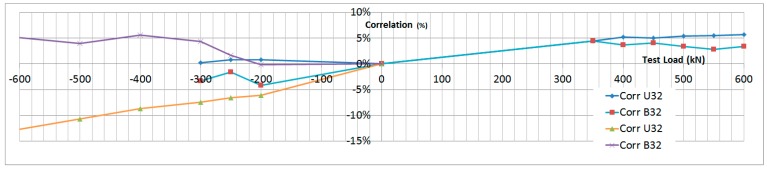
Failure Load Test Data Correlation.

The data corresponding to the failure load test sequence is shown in Figure 19 and Figure 20. The data corresponding to the Limit Load sequences is shown as well for reference.

In conclusion, the correlation between the strain gauge data and the OF sensors is very high in the LL range (±5%) and it decays slightly as the failure load of the specimen is approached, which is related to the non-linear behavior of the specimen at this high load level and the fact that location accuracy of the sensors becomes more critical.

### 6.2. Strain Measurement along the OF

**Figure 21:**
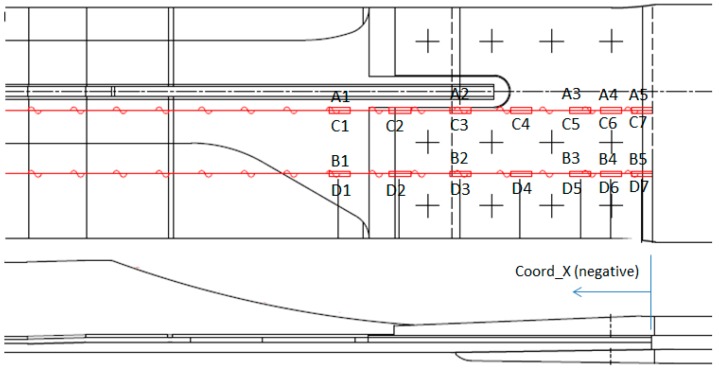
OF Sensor Location.

This paragraph analyzes the measurement along the OF lines carried out at Limit Load in Compression (−300 kN) and Tension (650 kN). The exact location of each sensor is shown in Figure 21. A and B FO lines are located on the upper surface of the specimen, on the stringer foot, while C and D are located on the lower surface.

**Figure 22:**
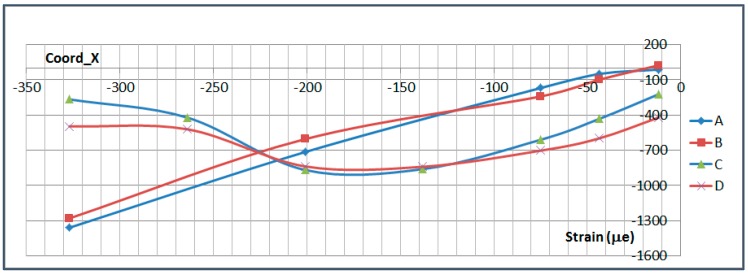
Strain Data along OF in Compression (−300 kN).

**Figure 23:**
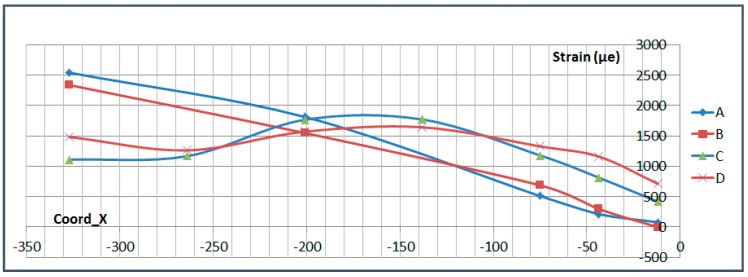
Strain Data along OF in Tension (650 kN).

Figure 22 and Figure 23 show the measurements along the span of the specimen (Coord_X) with each sensor couple (A/C or B/D) corresponding to a different chordwise (Coord_Y) position. The *X* coordinate of each sensor is indicated in Table 6. Coord_Y is measured from the Stringer Web centre line.

These two plots show a slight chordwise difference due to the influence of the stringer web and specimen edge proximity and a more remarkable curvature of the specimen under compression load which is consistent with the plots shown in the previous paragraph and with the fact that compression tends to increase the initial eccentricity of the specimen due to the Stringer Run-out geometry while tension tends to straighten it.

Two interesting conclusions can be extracted from this graphs:

- Firstly, thanks to the very low distortion produced by the OF installation, measurements have been possible up to the edge of the specimen under the bolted joint with the metallic plates.
- These measurements show how the composite part is gradually unloaded as the load is transferred to the metallic elements through the bolting. This effect will be further analyzed in more detail in Section 6.4.

Curvature readings can be extracted from the OF readings as well. This aspect is analyzed in the following paragraph.

### 6.3. Curvature Analysis

The curvature analysis of the specimen can be made by comparing the difference between the upper and lower OF sensor along the span of the specimen. The formula to obtain the curvature is:

(4) $$Curv=\frac{1}{R_{Curv}}=\;\frac{\varepsilon_{TOP}-\varepsilon_{BOTTOM}}{Thickness}$$

where *R_Curv_* is the curvature radius, *ε_TOP_* is the strain on the top surface and *ε*_BOTTOM_ on the botton surface_._

The thickness of the specimen is not perfectly constant along the span but the difference has been disregarded in this analysis.

**Figure 24:**
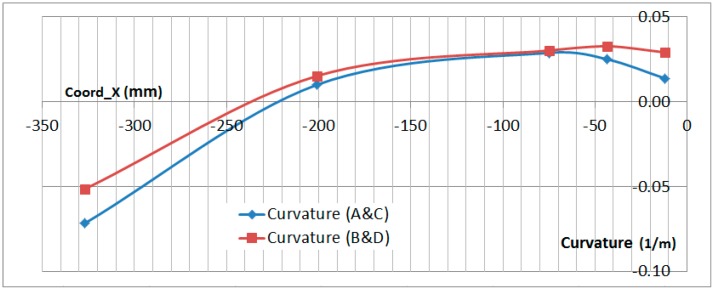
Calculated Curvature in Compression (−300 kN).

**Figure 25:**
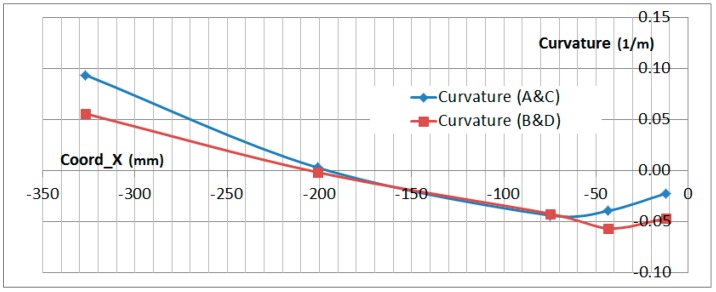
Calculated Curvature in Tension (650 kN).

Figure 24 and Figure 25 show the curvature of the two OF lines along the stringer *X* coordinate. This measurement has been obtained in the bolting area up to the edge of the composite part with no disturbance on the interface with the metallic plates and provides a very useful insight of the secondary bending produced on the specimen. Curvature measurements are only provided on those locations where both readings on the upper and lower surface exist.

Figure 24 and Figure 25 show that the curvature reached under compression load equals the value (in absolute value) of the curvature reached in tension. Considering that the absolute value of the compression test load is half the value of the tension load, this data clearly shows the tendency of the compression load to magnify the bending effect of the geometrical eccentricity of the Stringer Run-out. 

**Figure 26:**
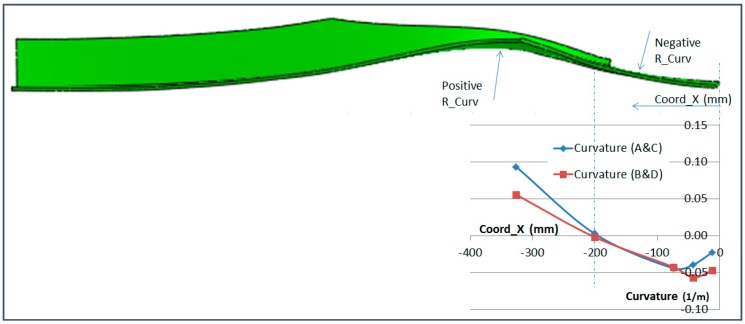
Predicted Specimen Deflection vs. OF Sensor Curvature.

The curvature data obtained from the OF sensors under tension load has been qualitatively compared with the FEA prediction for the test in Figure 26. The *X* axis of the graph has been scaled to match the size of the specimen.

This final figure shows the good correlation between the curvature obtained from the OF sensors and the prediction from a FEA model carried out for the test. Especially considering the inflection point which is produced in the Stringer Run-out Area. The deflected shape shown in Figure 26 is not to scale.

### 6.4. Analysis of the Load Transfer in the Bolting Area

The total load running from the composite part into the metallic parts through the bolting area has been analyzed in this paragraph. The fact that the composite part is instrumented in the bolting area allows the effect of the load transfer at the joint to be shown. 

The load in the composite part at each section has been calculated from the OF sensors placed on the upper and lower surface. The load has been obtained considering the average elastic modulus of the composite part and the thickness of each instrumented section.

The load in the metallic part has been obtained from the data of the strain gauge sensors which are placed on the upper surface of the triform (upper metallic part) and the lower surface of the buttstrap (lower metallic part), assuming that the average strain is representative of the strain of both parts. Then the elastic modulus of the parts and their geometry have been considered.

**Figure 27:**
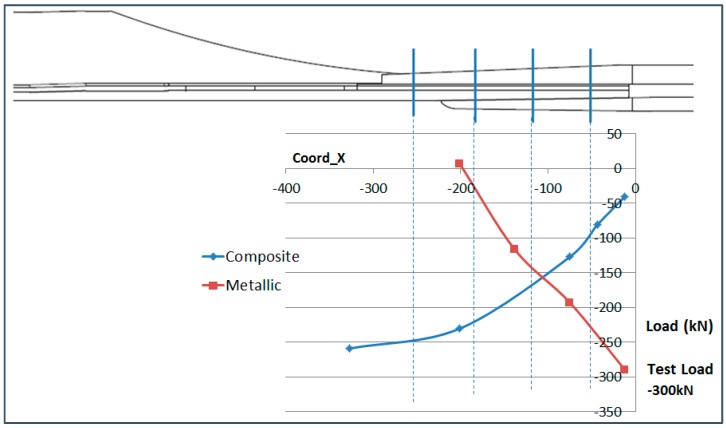
Load Split in Compression (−300 kN).

**Figure 28:**
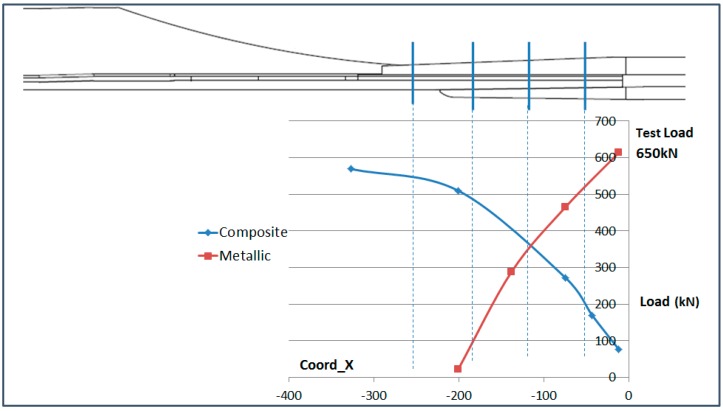
Load Split in Tension (650 kN).

The result of this analysis is shown in Figure 27 for the Compression load and Figure 28 for the Tension load. The vertical blue lines indicate the position of each row of bolts. The calculations made in this paragraph have considered average strain on the structural elements and therefore, the values obtained are approximate but they allow the visualization of the load transfer between the structural elements.

The total test load has been indicated in the graphs. It is noteworthy that the composite part (blue line) does not seem to recover the full running test load as it reaches a maximum of around 85%. This is due to the fact that the OF sensors only measure the load running through the skin and the stringer foot and therefore, the load running through the stringer web is not accounted for in these graphs.

## 7. Conclusions

This work has presented and demonstrated a methodology to successfully integrate embedded optical fiber sensors and connectors for strain monitoring purposes in a composite structural part representative of the root joint area of the wing lower cover of a real aircraft. The process was prepared and carried out under the main premise of being compatible in the production phase and ensuring the subsequent performance of sensors and connectors in the structural test phase.

It can be considered one of the first, if not the first, demonstration of the integration of embedded optical fiber sensors in a composite wing structure during production in an aeronautical composite plant.

Trimmable optical fiber connectors were specifically designed and manufactured for this real application and have enabled the trimming of the structure after autoclave curing process and the robust interrogation of the embedded FBG during the structural tests. 

The analysis of the strain results have demonstrated good correlation between fiber sensors and electrical strain gauges in those locations where they are installed nearby.

The curvature analysis of the specimen in the stringer run out and the load transfer analysis in the bolting area have demonstrated the consistency of optical fiber sensor results after being compared with the predictions from an FEA model carried out for the test.

To sum up, the methodology and results of these tests can be considered strong evidence of the satisfactory integration of embedded optical fiber sensors inside the automatic tape layout process during production and definitely contributes to the serious consideration of embedded optical fiber sensors for serial composite structural test applications in the near future.

## Figures and Tables

**Figure 8:**
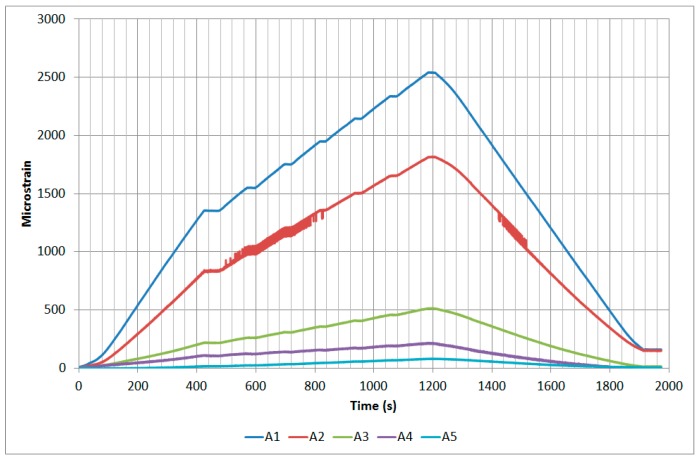
Specimen 1-Root joint -Before fatigue -tensile LL- Channel A.

**Figure 9:**
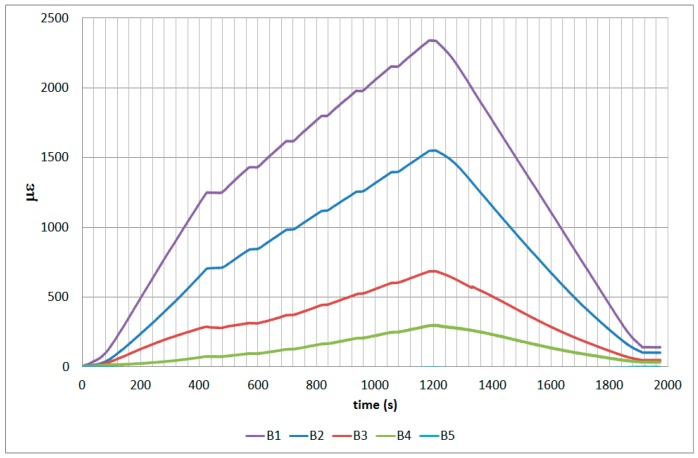
Specimen 1-Root joint -Before fatigue -tensile LL- Channel B.

**Figure 10:**
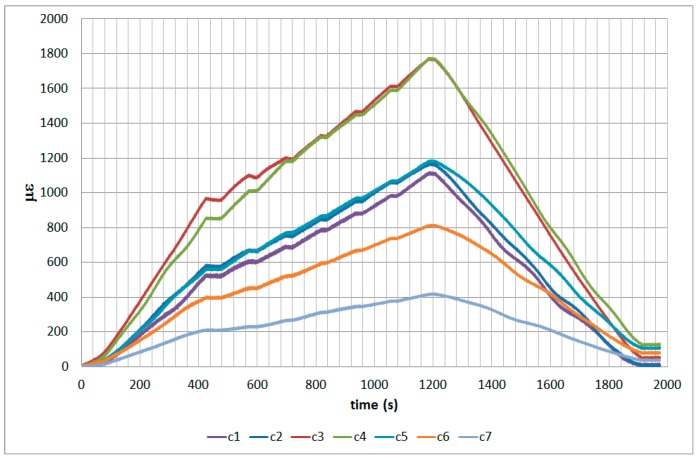
Specimen 1-Root joint -Before fatigue -tensile LL- Channel C.

**Figure 11:**
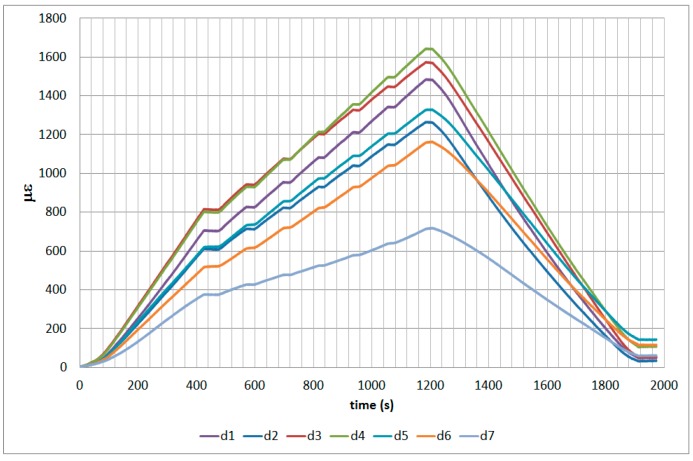
Specimen 1-Root joint -Before fatigue -tensile LL- Channel D.

**Figure 12:**
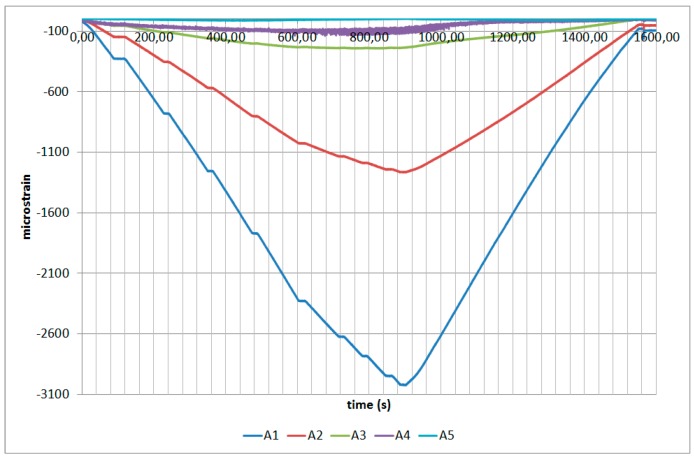
Specimen 1-Root joint -After fatigue -compression UL- Channel A.

**Figure 13:**
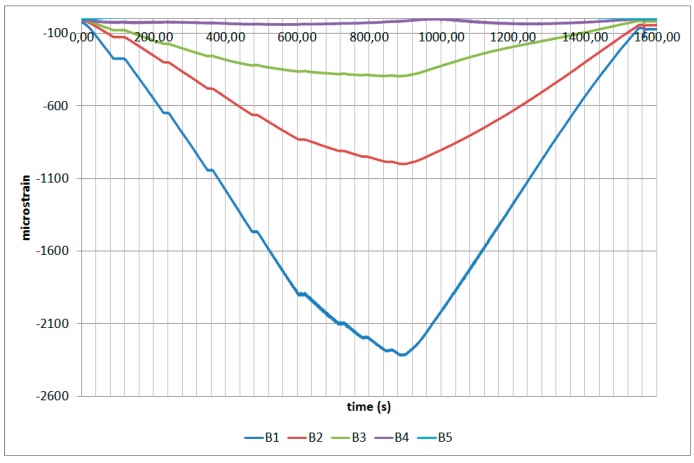
Specimen 1-Root joint -After fatigue -compression UL- Channel B.

**Figure 14:**
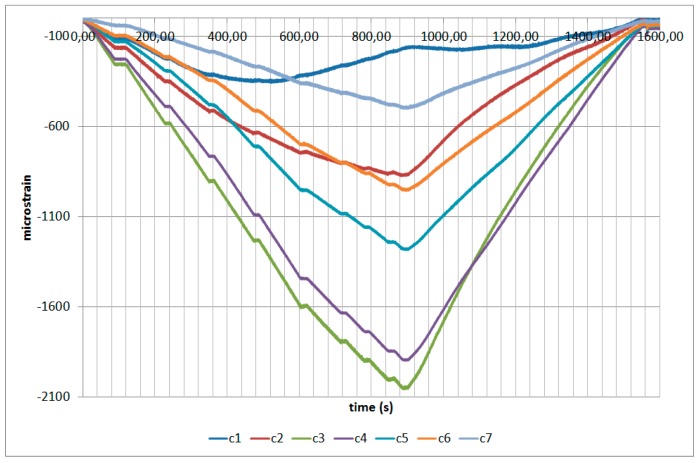
Specimen 1-Root joint -After fatigue -compression UL- Channel C.

**Figure 15:**
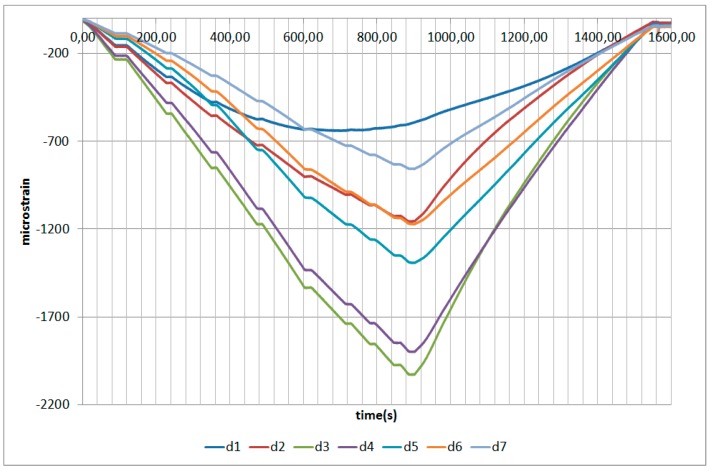
Specimen 1-Root joint -After fatigue -compression UL- Channel D.

**Figure 16:**
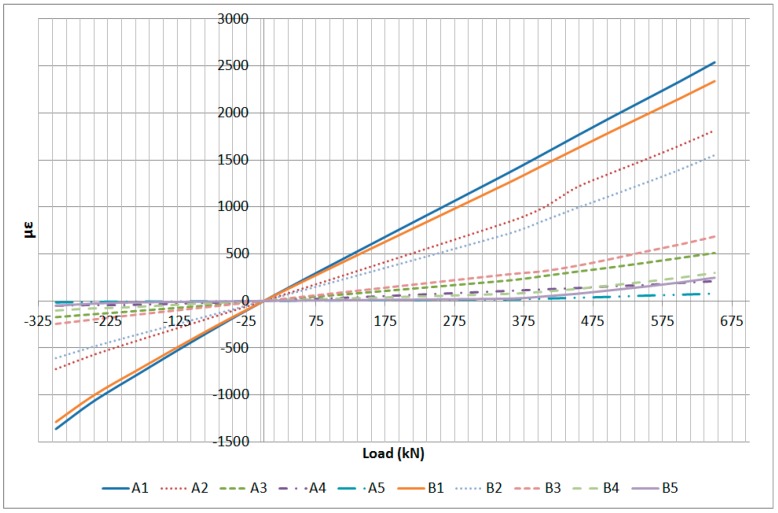
Specimen 1- FBGs embedded on stringer foot-tensile & compression after fatigue.

**Table 1:** Main features of the FBG arrays.

Parameters	Fiber Array	
	OF1	OF2
Written process	DTG (Draw Tower Grating)	
Coating	ORMOCER	
Coated fiber diameter (µm)	200	
FBG length (mm)	10	
FBG reflectivity (%)	>15	
FWHM (pm)	100	
N° FBG/fiber	5 (1–5)	7 (1–7)
Wavelength center (nm)	1531, 1553, 1564, 1570 and 1576	1520, 1531, 1542, 1553, 1564, 1570 and 1576
Operational temperature	−180 °C to 200 °C	

**Table 2:** Limit Load Test Data.

	Optical Fiber Sensors				Strain Gauges	
	A1	B1	C1	D1	U32	B32
Load (kN)	με	με	με	με	με	με
−300	−1360	−1281	−267	−497	−1317	−395
−250	−1090	−1027	−249	−418	−1050	−339
−200	−860	−808	−208	−343	−828	−287
0	0	0	−2	1	0	0
350	1354	1252	523	706	1246	588
400	1552	1434	602	828	1417	689
450	1752	1619	695	955	1602	792
500	1948	1800	781	1082	1776	901
550	2143	1980	877	1213	1951	1016
600	2337	2155	982	1344	2122	1124
650	2542	2338	1109	1483	2307	1246

**Table 3:** Limit Load Correlation.

	Correlation U32		Correlation B32	
Load (kN)	Avg. (A1, B1)	%	Avg. (C1, D1)	%
−300	−1320	0.2%	−382	−3.3%
−250	−1058	0.8%	−334	−1.6%
−200	−834	0.8%	−275	−4.2%
0	0	-	−1	-
350	1303	4.5%	615	4.4%
400	1493	5.2%	715	3.7%
450	1685	5.1%	825	4.1%
500	1874	5.4%	932	3.4%
550	2062	5.5%	1045	2.8%
600	2246	5.7%	1163	3.4%
650	2440	5.6%	1296	3.9%

**Table 4:** Failure Load Test Data.

	Optical Fiber Sensors				Strain Gauges	
	A1	B1	C1	D1	U32	B32
Load (kN)	με	με	με	με	με	με
0	0	−2	−7	1	0	0
−200	−970	−806	−264	−396	−944	−330
−250	−1253	−1041	−306	−477	−1225	−385
−300	−1566	−1300	−338	−542	−1543	−421
−400	−2198	−1805	−336	−625	−2183	−454
−500	−2621	−2094	−267	−638	−2625	−435
−610	−3018	−2318	−169	−602	−3037	−366

**Table 5:** Failure Load Correlation.

	Correlation U32		Correlation B32	
Load (kN)	Avg. (A1, B1)	%	Avg. (C1, D1)	%
0	−1	-	−3	-
−200	−888	−6.1%	−330	−0.1%
−250	−1147	−6.6%	−392	1.7%
−300	−1433	−7.4%	−440	4.4%
−400	−2002	−8.7%	−480	5.6%
−500	−2358	−10.7%	−453	4.0%
−610	−2668	−12.9%	−386	5.2%

**Table 6:** OF Sensor Locations.

	Coord_Y (mm)	Z Position	Coord_X (mm)						
			−327	−264	−201	−138	−75	−43.5	−12
**OF Line A**	19.5	Top	A1	-	A2	-	A3	A4	A5
**OF Line B**	85.5	Top	B1	-	B2	-	B3	B4	B5
**OF Line C**	19.5	Bottom	C1	C2	C3	C4	C5	C6	C7
**OF Line D**	85.5	Bottom	D1	D2	D3	D4	D5	D6	D7
